# High pressures and asymmetrical stresses in the scoliotic disc in the absence of muscle loading

**DOI:** 10.1186/1748-7161-2-4

**Published:** 2007-02-24

**Authors:** Adam R Meir, Jeremy CT Fairbank, Deborah A Jones, Donal S McNally, Jill PG Urban

**Affiliations:** 1 Nuffield Department of Orthopaedic Surgery, Oxford University, Oxford, UK; 2 Physiology Laboratory, Oxford University, Oxford, UK; 3 Institute of Biomechanics, University of Nottingham, Nottingham, UK; 4 Adam R Meir, co Dr. Jill Urban, Physiology laboratory, Oxford University, Oxford, UK

## Abstract

**Background:**

Loads acting on scoliotic spines are thought to be asymmetric and involved in progression of the scoliotic deformity; abnormal loading patterns lead to changes in bone and disc cell activity and hence to vertebral body and disc wedging. At present however there are no direct measurements of intradiscal stresses or pressures in scoliotic spines. The aim of this study was to obtain quantitative measurements of the intradiscal stress environment in scoliotic intervertebral discs and to determine if loads acting across the scoliotic spine are asymmetric. We performed in vivo measurements of stresses across the intervertebral disc in patients with scoliosis, both parallel (termed horizontal) and perpendicular (termed vertical) to the end plate, using a side mounted pressure transducer (stress profilometry)

**Methods:**

Stress profilometry was used to measure horizontal and vertical stresses at 5 mm intervals across 25 intervertebral discs of 7 scoliotic patients during anterior reconstructive surgery. A state of hydrostatic pressure was defined by identical horizontal and vertical stresses for at least two consecutive readings. Results were compared with similar stress profiles measured during surgery across 10 discs of 4 spines with no lateral curvature and with data from the literature.

**Results:**

Profiles across scoliotic discs were very different from those of normal, young, healthy discs of equivalent age previously presented in the literature. Hydrostatic pressure regions were only seen in 14/25 discs, extended only over a short distance. Non-scoliotic discs of equivalent age would be expected to show large centrally placed hydrostatic nuclear regions in all discs. Mean pressures were significantly greater (0.25 MPa) than those measured in other anaesthetised patients (<0.07 MPa). A stress peak was seen in the concave annulus in 13/25 discs. Stresses in the concave annulus were greater than in the convex annulus indicating asymmetric loading in these anaesthetised, recumbent patients.

**Conclusion:**

Intradiscal pressures and stresses in scoliotic discs are abnormal, asymmetrical and high in magnitude even in the absence of significant applied muscle loading. The origin of these abnormal stresses is unclear.

## Background

The aim of this study was to obtain measurements of pressures and stresses in the intervertebral discs of scoliotic patients in order to try to prove the hypothesis that asymmetrical loading was present in the scoliotic spine and hence could realistically be involved in curve progression. We were also very interested in the stress environment of the intervertebral disc in scoliosis in general. We considered that recording from discs prior to excision during anterior scoliosis reconstructive surgery involved minimal additional patient risk. However, in anaesthetised patients with additional muscle relaxation (an essential aspect of the anaesthetic procedure) no loading due to muscle activity is present. We therefore were unsure if our approach would show any meaningful results.

Previous authors have theorised about the biomechanical role of asymmetrical loading in progression of the scoliotic deformity [1-3]. Non-operative treatments such as bracing are designed to counteract these loads [4-6]. The majority of studies have focused on the musculature as the origin of this loading asymmetry. Electromyographic measurements have demonstrated differences in muscle activity between the convex and concave sides of the spine [7-9]. Muscle biopsies additionally find a significantly lower percentage of Type I fibers in the multifidus muscle on the concave side, particularly at the curve apex and also in the superficial muscles above and below the apex [10-13].

The cellular mechanisms by which abnormal loading may generate deformity has been shown previously in cell culture and in animal models. Cells of bone and disc are very responsive to mechanical stress[14,15], with high loads inhibiting longitudinal growth [16-18]. An asymmetric load may thus lead to asymmetrical longitudinal growth and hence wedging of the vertebral bodies and intervertebral disc[19]. Scoliotic-like deformities have also been produced in otherwise healthy animals by applying asymmetrical loads across the spine [19-23]. These loads differentially affect longitudinal bone growth. The disc remodels and becomes wedged and distorted. Thus asymmetrical loading could lead to permanent changes in vertebral bodies and discs and hence contribute to progression of the scoliotic deformity.

Most of the information on loads acting on the scoliotic spine is derived from measurements of changes in spinal morphology and from modelling[3,24]. There have however been no direct measures of loading asymmetry. We have therefore used stress profilometry [25] to directly measure pressure and stress profiles across scoliotic discs during anterior reconstructive surgery. This technique involves introducing a miniature pressure transducer, side mounted on a needle, across the disc transversely. Two linear profiles of stresses are measuring with the transducer orientated parallel (termed horizontal) and perpendicular (termed vertical) to the endplate.

## Materials and methods

### Patient selection

Patients having anterior surgery for thoraco-lumbar scoliosis were considered for inclusion in this study. The control group was patients without scoliosis having anterior surgery involving total disc excision. These were patients undergoing kyphosis surgery or anterior lumbar surgery for back pain. They were chosen as a comparison group to the scoliotics because of the lack of lateral curvature at the disc levels operated on. Ethical approval was obtained from the Oxford Regional Ethics Committee. Written and verbal information about the study was given to patients 4–6 weeks before surgery. In those patients under the age of 16 years, the parents' consent was obtained as well. Details of the patients and controls tested are given in tables 1 and 2.

**Table 1:** Details of Scoliotic patients examined.

**Patient no.**	**Age years**	**Sex**	**Diagnosis**	**Curve type**	**Cobb angle**	**Apical disc level**	**Surgical approach**	**Disc levels recorded**
1	16	F	Idiopathic	L Thoracolumbar	55	T12/L1	L. Thoraco-lumbar	T11/12, **T12/L1**, L1/2
2	17	F	Idiopathic	L. Thoracolumbar	78	T12/L1	L. Thoraco-lumbar	T11/12, **T12/L1**, L1/2
3	13	F	Neuro-muscular	L. Thoracolumbar	60	L1/L2	L. Thoraco-lumbar	T12/L1, **L1/2**, L2/3, L3/4
4	24	F	Idiopathic	R Thoracolumbar	44	T11/12	R. Thoraco-lumbar	T10/11, **T11/12**, T12/L1
5	15	M	Idiopathic	R Thoracolumbar	57	T11/12	R. Thoraco-lumbar	T10/11,**T11/12**, T12/L1
6	25	F	Idiopathic	L. Thoracolumbar	63	T10/11	L. Thoraco-lumbar	T9/10, **T10/11**, T11/12, T12/L1.
7	24	F	Idiopathic	R. Thoracolumbar	59	L1/2	R. Thoraco-lumbar	T11/12, T12/L1, **L1/2**, L2/3, L3/4

**Table 2:** Details of non-Scoliotic Patients (Control) examined.

**Patient no.**	**Age years**	**Sex**	**Diagnosis**	**Clinical detais**	**Surgical approach**	**Disc levels recorded**
8	18	F	Neuromuscular kyphosis	Fibrillary Astrocytoma. Paraplegia 3 years.	R. Thoraco-lumbar	T11/12, T12/L1, L1/2
9	15	F	Thoracic Kyphosis	Idiopathic. Curve present since age 3.	L thoracotomy	T3/4, T4/5, T6/7, T7/8
10	26	F	Discogenic back pain L 4/5	14 y low back pain. Provocative discography positive L4/5	L. Retroperitoneal	L4/5
11	40	F	Discogenic back pain L4/5, L5/S1	Low back and right leg pain. MRI showed mild degeneration L4/5 and 5/S1.	L. Retroperitoneal	L4/5, L5/S1

### Pressure measurements

#### Pressure transducer

The pressure transducer used in the study was the same type as that used previously for pressure profile measurements [25-28]. It has been shown to give reproducible results when used in human intervertebral discs[25,26]. The device consisted of a pressure-sensing diaphragm, side mounted 3 mm from the tip of a 10 cm long, 1.33 mm diameter blunt-ended surgical steel needle (AISI type 304, Gaeltec Ltd, Dunvegan, Isle of Skye, UK). A millimetre scale marked additionally at centimetre intervals was etched onto the needle to allow measurement of depth of penetration of the needle. A small metal bar was added to the base of the needle to enable the surgeon to orientate the transducer diaphragm. The transducer was connected to an isolated amplifier (ADInstruments, Oxford UK) which as well as amplifying the signal from the sensor also provided a 5 V excitation signal. A laptop computer running Chart software (ADInstruments, Oxford UK) interfaced with a PowerLab data acquisition system (ADInstruments, Oxford UK) was used to record and analyse the data.

The transducer was calibrated up to 1 MPa using a pressure vessel(a tube of circular cross section) with a known cross sectional area filled with water to which known loads were applied by a servocontrolled hydraulic materials testing machine (Dartec, Dartec Ltd. Stourbridge, United Kingdon). The rate of application of load during calibration was approximately 0.2 MPa/s. Over the range 0–1 MPa the voltage recorded was found to be linear with respect to pressure applied. Re-calibrations of the transducer showed that it was very stable and showed negligible drift over time. Before surgical use, the pressure transducer was sterilised using Perisafe (Antec International, Sudbury, UK) according to standard hospital protocols.

#### Stress profilometry

**Figure 1:**
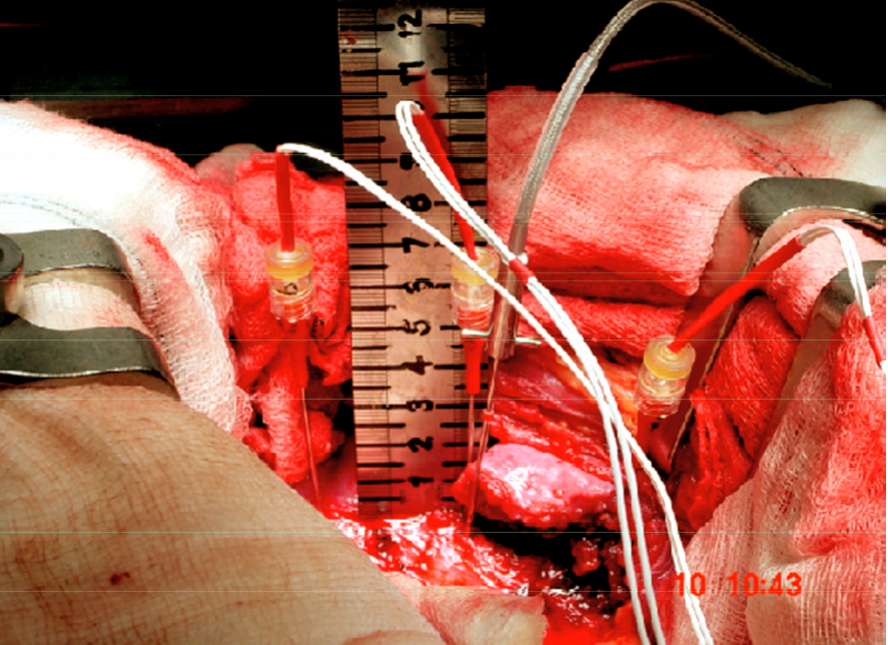
**Photograph of intraoperative measurement set up**. Rostral is to the left, caudal to the right. Three electrodes can be seen inserted through the convex annulus of three adjacent discs. The pressure transducer is seen inserted into the middle disc.

**Figure 2:**
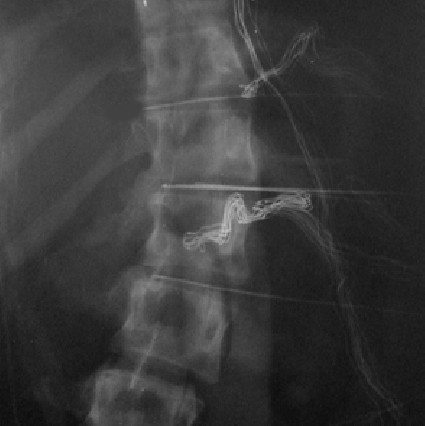
Part of intraoperative representative radiograph showing electrodes and pressure transducer inserted from the convex side of the scoliotic curve (same patient as figure 1).

**Figure 3:**
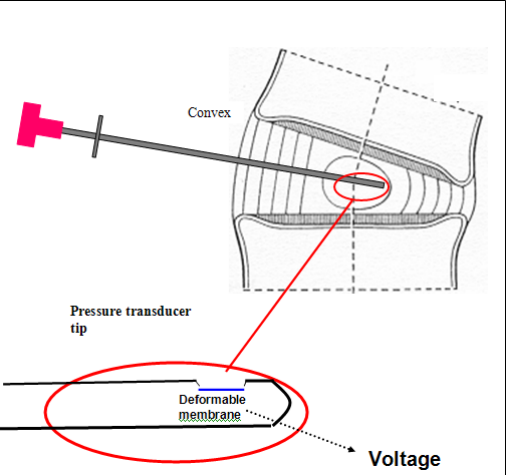
**Diagram of position of needle mounted pressure transducer in scoliotic disc during intraoperative pressure measurements**. Detail of pressure transducer tip included to show plane of transducer membrane parallel to axis of introducer needle.

In all the scoliosis and kyphosis cases the spine was exposed by a conventional transpleural and retroperitoneal approach, dividing the diaphragm close to its peripheral attachment. In the back pain patients, a retroperitoneal approach to the low lumbar spine was performed. Our normal practice is to insert spinal needles into exposed discs and to check the anatomical level by taking an x-ray. In these scoliosis and control patients, one of the needles was substituted by the pressure transducer. It was inserted through the convex annulus and the transducer pushed across the disc to the concave annulus (Figure 1), the path of the needle approximating to the coronal plane (for the control back pain discs, the transducer path was in the antero-posterior or Sagittal plane). A marker X-ray film was then taken and used to determine the position of the pressure transducer within the disc (Figure 2). In the time taken to develop the marker film (~7 minutes), stress profiles were measured as described previously[25]. Stress readings were obtained at 5 mm intervals by withdrawing the transducer manually (Figure 3). At each position, stresses were recorded with the sensor membrane orientated both towards (vertical stress) and at 90° (horizontal stress) to the endplate. This profile was repeated in the other exposed discs where the transducer was aligned with each of the previously placed needles. The disc levels chosen for stress analysis were selected by those discs that would definitely be removed as part of the anterior surgical release. This ensured that no discs remaining after surgery would be potentially damaged by the measurement procedure.

#### Analysis of data

The initial position of the transducer/electrode tip and of the transducer membrane in each disc was assessed from the intra-operative X-ray film. These were scaled using the known diameter and length of the pressure transducer. The disc dimensions were obtained from the same X-ray. Results are given as the position of the transducer diaphragm relative to the concave border of the disc.

Chart software (ADInstruments, Oxford, UK) was used to convert the voltages obtained into stresses/pressures in accordance with the measured calibration coefficient. The data were then exported to Microsoft Excel for further analysis.

### Statistical analysis

Where appropriate, measurements are presented as means and standard deviations in n discs where n is the number of discs tested.

## Results

### Patient details

In this study, pressure profiles were measured in 25 scoliotic and 10 non-scoliotic discs from 7 scoliotic and 4 non-scoliotic patients over a period of one year (July 2001 – July 2002). Details of the scoliotic patients in the study are given in Table 1 and of the non-scoliotic patients in Table 2. The age range of the scoliotic patients was from 13 to 25 years (mean age 19.1 ± 5.0 years) and of the non-scoliotic patients was 15–40 yrs. The majority of scoliotics were female (6/7). In 6/7 patients there was no obvious underlying cause for the curve, classified as idiopathic; the one neuromuscular (patient 3) was severely disabled with microcephaly, epilepsy and possible Rett's syndrome. The Cobb angle of the curves varied from 44 to 78 degrees, mean 59.4 ± 10.1. There were four left and three right sided thoracolumbar curves and the apical disc was at T11/T12 or T12/L1 in 4/7 patients. Two of the non-scoliotic patients were treated for kyphosis and two for back-pain.

## Scoliotic discs

### Patterns of stress profiles seen across scoliotic discs

The stress profiles generally showed asymmetries between the concave and convex sides of the curve for both the vertical and horizontal stresses.

**Figure 4:**
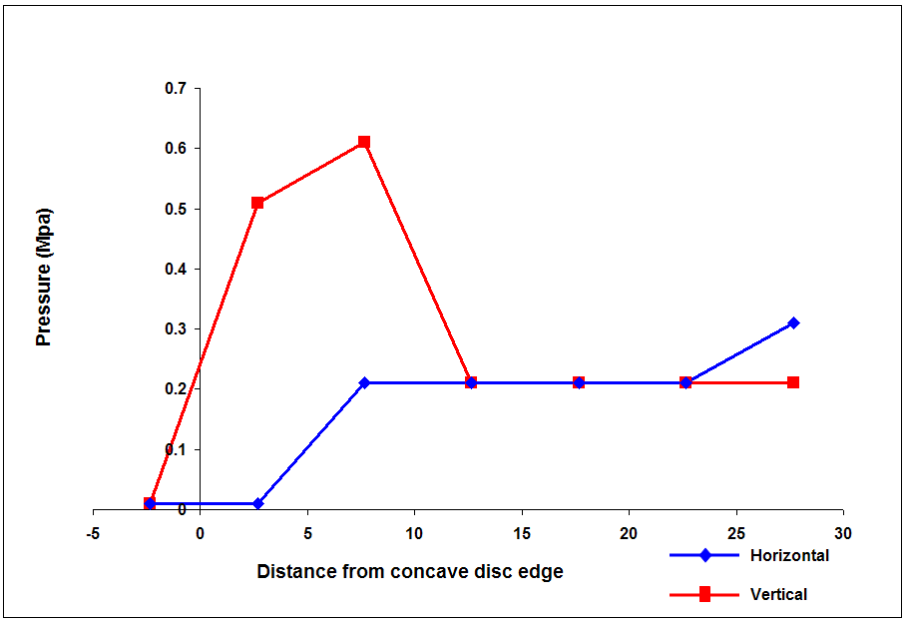
**Typical profile of measurements across a single disc with a hydrostatic region**. These results showing the typical features of a vertical stress peak in the concave annulus and a hydrostatic region towards the convexity were recorded in the L1/L2 disc of a 13 year old neuromuscular scoliotic patient (Patient 3).

A peak in the vertical stress recorded in the concave annulus (far left side of the profile) was a common feature of the profiles in the scoliotic discs in this study, a typical example being shown in figure 4. This shows the vertical and horizontal stress profiles recorded during anterior scoliosis surgery in the L1/2 disc of patient 3, a 13 year old neuromuscular patient. When stress peak is defined as a stress value of at least twice that recorded in the anatomical nucleus, 13/25 scoliotic discs have this feature.

**Figure 5:**
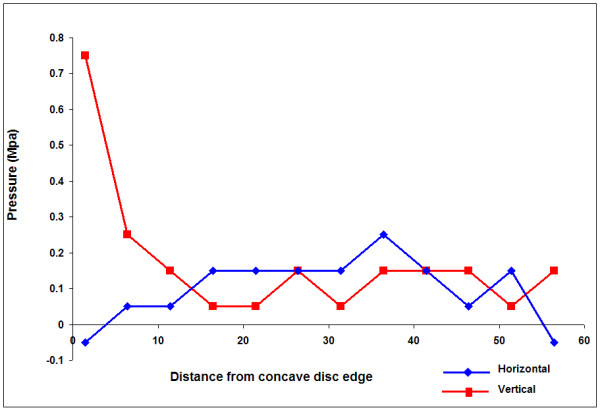
**Typical profile of measurements across a single disc with no hydrostatic region**. These results showing a vertical stress peak at the concavity but no hydrostatic region were recorded in the L1/L2 disc of a 17 year old idiopathic scoliotic patient (Patient 2).

Previous pressure profiles measured in non-scoliotic, non-degenerate discs of similar age to the scoliotic patients show large hydrostatic central areas (equal vertical and horizontal pressures)[25,26] In many scoliotic discs we found that over significant areas, horizontal and vertical stresses recorded from the centre of the disc were of different magnitude indicating non-hydrostatic behaviour. In order to quantify this, we defined a hydrostatic region as one in which over at least two consecutive readings, the horizontal and vertical stresses were within 0.01 MPa. Infact, in 11/25 discs there were no regions of hydrostatic pressure. A disc with a hydrostatic region is illustrated in figure 4, the hydrostatic pressure region is shown at consecutive readings 13,18 and 23 mm from the concave disc boundary and can be seen to be shifted towards the convex side of the nucleus. An example of a disc with no hydrostatic regions is shown in figure 5 (Patient 2 L1/L2, 17 year old with idiopathic scoliosis) which also shows the pattern of high vertical stresses in the concavity compared to other regions of the disc.

In 5 cases, peaks in the convex annulus also occurred. On only 2 occasions was this higher than the maximum vertical stress in the concave annulus in the same disc.

### Disc hydrostatic pressures

**Figure 6:**
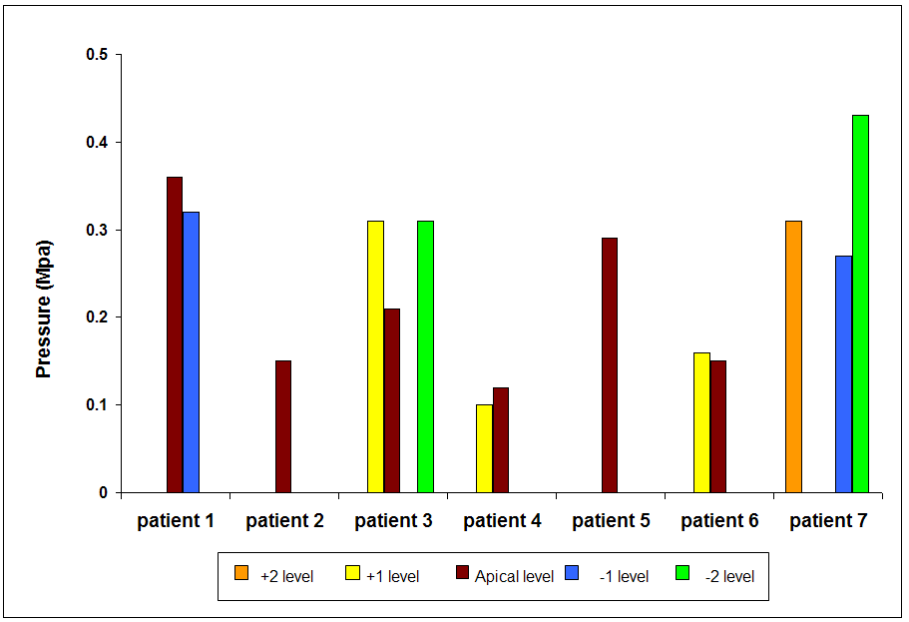
**Hydrostatic pressure levels measured in all scoliotic discs where a hydrostatic pressure region was recorded**. Results are shown versus disc level relative to the apical disc.

We analysed the pressures recorded in those scoliotic discs that showed hydrostatic regions. The hydrostatic pressure values varied between 0.1 and 0.43 MPa; the mean value was 0.25 ± 0.10 MPa (SD). The values for each patient in relation to the apical disc are shown in figure 6. On the abscissa, positive levels denote rostral and negative levels caudal location relative to the curve apex.

Hydrostatic disc pressures in the apical disc were not obviously higher overall or higher than at adjacent levels and the apical discs had more hydrostatic nuclei 6/7 (86%) compared with 10/18 non-apical discs (56%).

**Figure 7:**
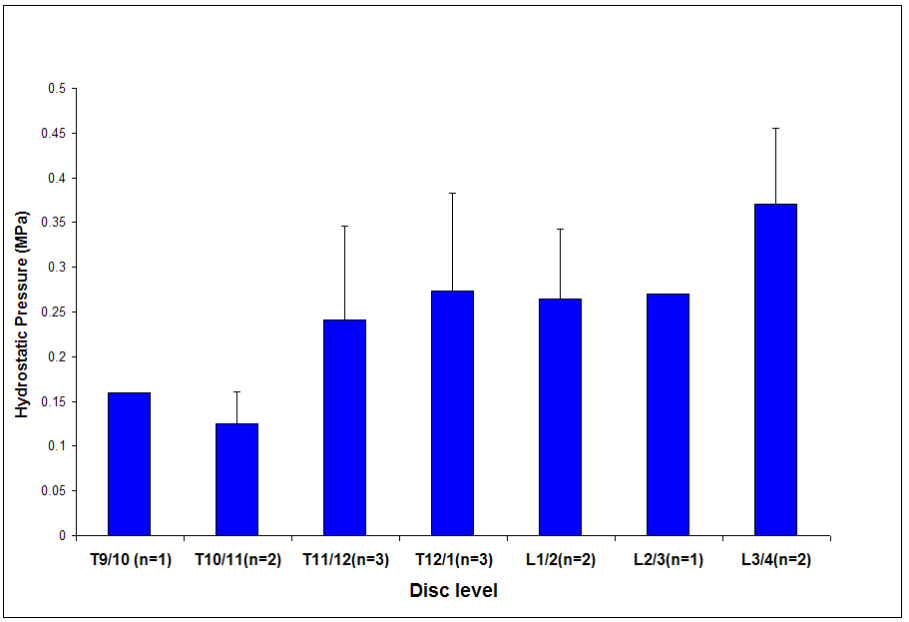
**Variation of the mean hydrostatic pressure level with absolute disc level**. Results shown for all scoliotic discs where a hydrostatic pressure region was recorded.

We found a slight trend towards higher mean pressure progressing caudally for absolute disc level, shown in figure 7. This graph shows the mean pressure at each disc level for all the scoliotic patients. Mean pressures were >0.1 MPa at all levels and tended to be similar in magnitude apart from the rostral 2 discs and most caudal disc. There was also no effect of age on levels of pressure measured (not shown).

### Maximum stress in the concave annulus

**Figure 8:**
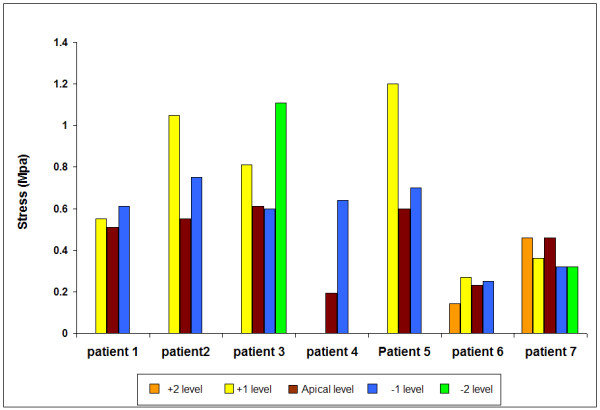
**The maximum vertical stress measured in the concave annulus in scoliotic discs**. Results are shown for all discs in which a stress peak was recorded versus disc level relative to the apical disc.

We analysed the maximum value of the stress in the concave annulus. No readings were obtained in the concave annulus of the T10/11 disc of patient 4 hence this disc is not represented. Stress levels were very variable, ranging from 0.1–1.15 MPa. For all discs where a recording was made in the concave annulus, the mean stress was 0.55 ± 0.28 MPa (SD), The magnitude of the peak vertical stresses recorded in the concave annulus for each of the scoliotic patients relative to apical disc level is shown in Figure 8. There was no consistent pattern from patient to patient. In 6/7 patients, the vertical stress recorded in the apical disc was less than that in adjacent discs.

### Stress change across the disc

The stress change across the disc was calculated from the first reading shown to be taken inside the anatomical disc minus the last reading taken before the transducer exited on the convex side. On some occasions, the last reading was probably out of the disc (both vertical and horizontal stresses suddenly fell to zero and these points were disregarded). In 18/24 discs, this value was positive indicating that higher stresses were found on the concave side of the curve. In only 2/7 patients was the stress positive and greater in the apical disc than adjacent discs. In 7/25 discs, stress was higher in the concave annulus than the convex without a concave stress peak being present.

**Figure 9:**
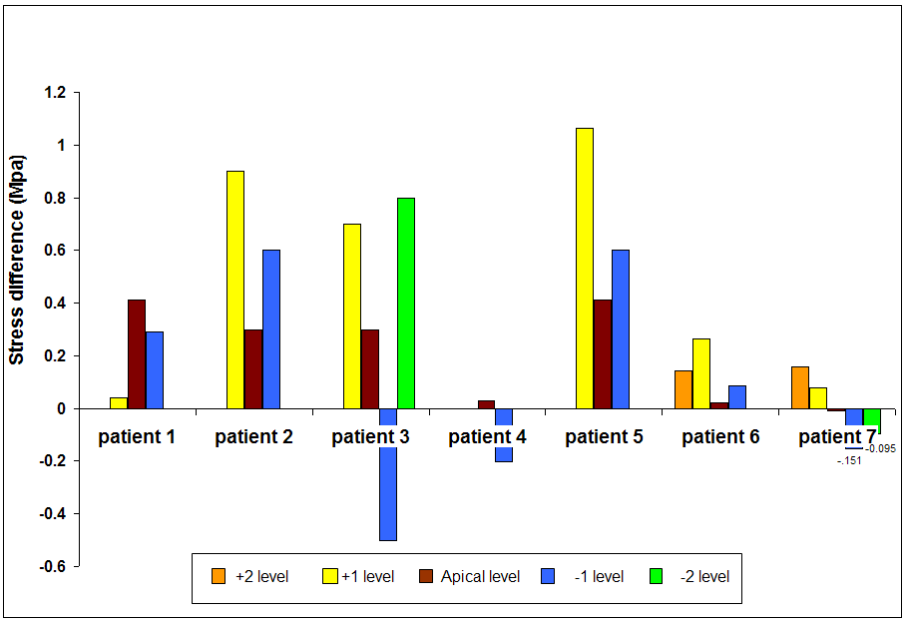
**Difference in vertical stress between the concave and convex annulus in scoliotic discs**. Results are shown for all discs where recordings were made in both the concave and convex annulus versus disc level relative to the apical disc.

The stress change across each of the scoliotic study discs is shown in Figure 9 relative to apical disc level; this difference gives a measure of load asymmetry.

## Non-scoliotic discs

Due to the low numbers of patients who both were having anterior surgery for non-scoliotic conditions and who consented to having pressure measurements performed during the time period of the study, we only have pressure profiles from 10 discs for comparison.

Table 2 shows clinical details for the two patients with back pain and two patients with kyphosis in the study.

### Patterns of stress profiles

Profiles showed significant variability in this group of patients.

Patient P10 who was 26 years old showed a pressure profile with higher stresses towards the posterior annulus, a hydrostatic region and a trough of horizontal pressure in the anterior annulus. In both discs of patient 11, stresses were essentially zero throughout apart from an increase in vertical stress in both and a horizontal stress peak in the anterior annulus of the L5/S1 disc with no hydrostatic regions.

In the kyphotic patients, patient 8 who had quite a flexible curve clinically showed profiles with pressure peaks mainly in the anterior annulus with an under pressurised central region. The T11/12 and L1/2 levels showed hydrostatic nuclei. Patient 9 who was a kyphotic with a curve that was noted to be very stiff intra-operatively had very narrow discs and made introduction of the pressure probe difficult. Only a few recordings per disc (mean of 2.75) were made and so profile shape could not be accurately determined.

### Hydrostatic pressures

5/10 (50%) non-scoliotic discs showed no regions of hydrostatic pressure.

For the patients with back pain, hydrostatic regions were seen in the L4/5 disc of patient 10 (0.214 MPa). The central regions of both discs in patient 11 showed negative pressures very close to zero level (L4/5 and L5/S1, means of -0.023 and 0.040 MPa respectively) and the horizontal and vertical pressures were very close in value over these regions. In the kyphotic patients, only the T11/12 and L1/2 discs of patient 8 showed hydrostatic regions with a mean of 0.030 ± 0.022 MPa. For patient 9, there were no hydrostatic regions however in order to indicate the general size of the stresses present, the mean vertical stress was 0.327 ± 0.33 MPa and horizontal was 0.627 ± 0.46 MPa over all 4 recorded discs.

### Stress peaks/stress change across non-scoliotic discs

5/6 (83%) non-scoliotic discs showed stress peaks in either the anterior or posterior annulus (data from Patient 9 was disregarded due to too few data points per disc) with the T11/12 disc of patient 8 showing both anterior and posterior peaks. Of these stress peaks, 5/7 (71%) were anterior.

**Figure 10:**
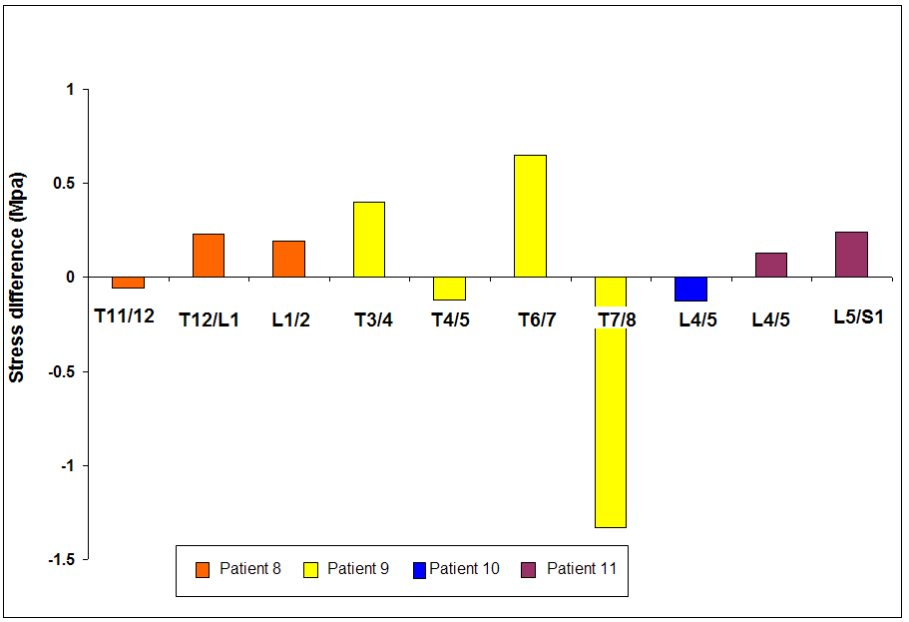
**Difference in vertical stress between the anterior and posterior annulus in non-scoliotic patients**. Results are shown versus absolute disc level for each disc.

All the non-scoliotic discs showed some stress difference across the disc. It was very variable in magnitude ranging from .057 to 1.33 MPa with a mean of 0.35 ± 0.39 MPa (See figure 10).

## Comparison of scoliotic and non-scoliotic hydrostatic pressures

Because of the small number of non-scoliotic patients in this study, with the permission of the author (Mr Yonezawa) we have also compared our results with intradiscal pressures measured in Japanese subjects with back pain prior to laser nucleotomy[29]. In this publication, intra-discal pressure measurements were made on patients under general anaesthesia and hence are comparable to our patients in terms of levels of spinal loading due to similar levels of muscular tension. This published series has older patients than those of the present study but neither we nor Yonezawa et al. found any effect of age on intradiscal pressure[29]We only used data from discs that we thought had hydrostatic nuclei from Yonezawa's study. We determined this by defining a hydrostatic nucleus as one in which the vertical and horizontal pressures differed by less than 0.01 MPa. For all those patients with hydrostatic nuclei (13/18) the mean disc pressure was 0.058 ± 0.03 MPa and for those age matched to the study patients the mean disc pressure was 0.070 ± 0.030 MPa.

In our study, the mean value of hydrostatic pressure for the scoliotic patients was 0.25 ± 0.10 MPa, > 3 fold higher than that measured in Yonezawa's patients.

**Figure 11:**
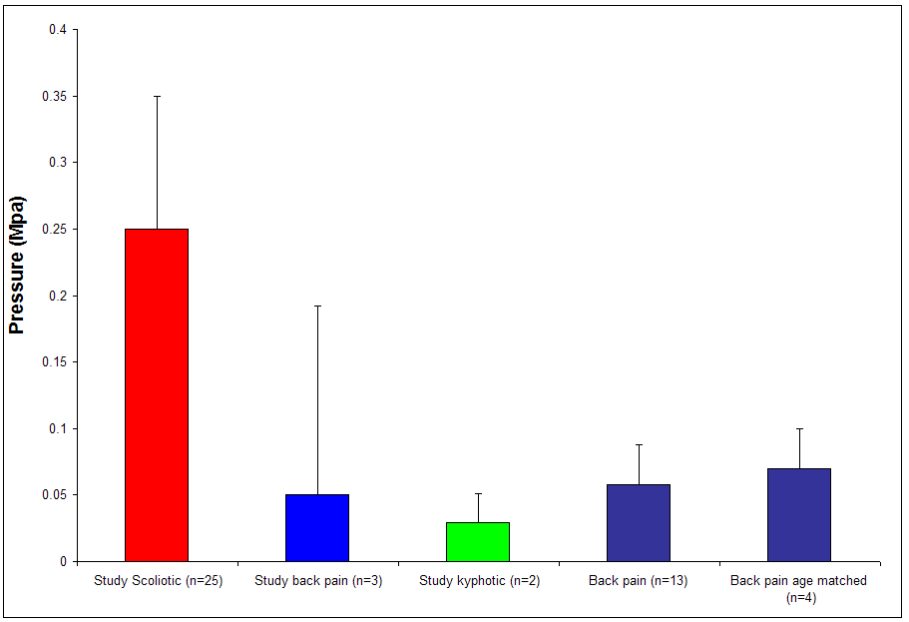
**Mean hydrostatic pressure measured during surgery in scoliotic discs compared to other conditions**. Details of the scoliotic and study back-pain and kyphotic patients are given in tables 1 and 2 respectively. The other results of disc pressures in patients with back pain are from the study of Yonezawa et al [29].

Figure 11 shows how the mean hydrostatic pressure measured in the scoliotic discs compared with pressures measured during surgery for other spinal pathologies. It is apparent that the pressure levels measured in scoliotic patients are considerably greater than those measured in other anaesthetized patients undergoing anterior spinal surgery

## Discussion

### Summary of results

We present new data on intra-disc pressure profiles of patients with thoraco-lumbar scoliosis. The overall hypothesis we were attempting to test in this study was that abnormal, asymmetrical loading was present in the scoliotic spine and contributed to the progression of the scoliotic deformity. However, due to the surgical positioning and anaesthetised state of the scoliotic subjects we were unsure whether any physiologically significant pressure readings would be obtained since the majority of loading across the motion segment is due to body weight and muscle activity [30-32]. We would perhaps have been expected to find pressures some way between 0.05 MPa and 0.14 MPa, the "intrinsic pressure" of cadaveric discs without muscle loading [33-36]. We also expected to find higher pressures, less hydrostatic discs and qualitatively different pressure/stress profiles in the apical disc than in adjacent discs since it is the most deformed and has more pronounced biochemical changes and lower cell viability than adjacent levels. [37-39].

Rather surprisingly, the readings obtained in the scoliotic discs were much higher in magnitude than we expected. The mean pressure reading in those discs with a hydrostatic nucleus (14/25) was 0.25 ± 0.10 MPa which was on average >3 fold greater than in the available control data (Figure 11).

Despite the lack of muscle activity, our results also showed evidence of asymmetrical loading in the scoliotic spine. Stresses were higher in the concave than in the convex annulus in 18/25 discs (Figure 9). We did not however find increased hydrostatic pressure (Figure 6) or peak stress levels (Figure 8) in the apical disc compared with its neighbours and in all but one disc, stress change was lowest in the apical disc compared with adjacent levels.

### Hydrostatic pressures

A number of scoliotic and non-scoliotic discs in this study had non-hydrostatic regions, 44% of scoliotic and 50% of non-scoliotic. For the nuclear matrix to behave in a non-hydrostatic manner it must be abnormal in composition or relatively dehydrated. This would be most likely due to glycosaminoglycan loss or potentially, mechanical loss of fluid due to chronic high loading. With regards to the non-scoliotic discs, the back pain patients would be expected to have degenerate discs with glycosaminoglycan loss. Patient 11 was known to have severe degeneration on MRI imaging and hence showed disc pressures close to zero and non-hydrostatic discs. Patient 10 was younger and had less severe degeneration consistent with higher pressures and a hydrostatic disc. For the kyphotic patients, patient 8 was a severely disabled, paraplegic from T4 distally due to invasion of the spinal cord by tumour. She had flexible kyphosis clinically and the T12/L1 level was non-hydrostatic presumably due to secondary degenerative change at that level. Patient 9 had a very stiff curve and almost complete loss of disc height at all levels and severely dehydrated discs. With regards to the scoliotic discs however, non-hydrostatic discs are less easily explainable. Glycosaminoglycan loss in not know to be severe in the scoliotic disc[37] and although the disc structure is abnormal, it is not classically degenerate [40-42].

The pressures we measured in discs with hydrostatic regions in the scoliotic patients were considerably higher than those measured in patients with kyphosis and with chronic low back pain, which were on average low and of similar magnitude to that reported by Yonezawa[29]. In addition, the mean intradiscal pressures measured in scoliotic patients (0.25 MPa; Fig 11) were also higher than pressures measured in healthy awake volunteers in similar postures, 0.12 MPa in the L4/5 disc of a healthy orthopaedic surgeon[43] and a mean of 0.15 MPa in a group of 22–29 y old Japanese volunteers with no disc degeneration[31]. These pressures indicate overloading of the scoliotic disc and hence if present chronically could cause relative dehydration and also lead to the non-hydrostatic behaviour seen in some discs.

The finding of high pressures and stresses in recumbent, anaesthetised scoliotic patients with minimal loading due to muscle activity was unexpected and its origins are unclear.

The internal mechanical environment of the intervertebral disc is complex. The factors influencing disc pressure/stress at any point in the disc will arise from both intrinsic factors, viz. disc swelling pressure and matrix organisation [44]and extrinsic factors including muscle action, body weight and ligamentous tethering[43]. Swelling pressure arises from the balance between tissue composition, particularly glycosaminoglycan concentration, and the opposing tension imposed by the collagen network [44]and while changes in glycosaminoglycan concentrations have been reported across scoliotic discs[37,45], these would tend to rather affect the swelling pressure profile than increase swelling pressure levels.

With regard to externally imposed forces, body weight and muscle forces, though altered in scoliotic patients[46], should play no role in these anaesthetized recumbent patients. The patients in this study were also all well supported inferiorly by an evacuated bean-bag which should have reduced externally imposed loading to a minimum. However, the surgical positioning of a patient with a curved, axially rotated spine might increase torsion in the deformed segments of the spine. The 7 surgical patients were all in the lateral position and in vitro tests show that imposition of rotation, flexion or extension in axially loaded spines can lead to a pressure rise in the disc [47,48].

Ligamentous tethering or changes in annulus organisation[49]could be another factor pre-stressing the disc and causing higher pressures however we feel that this is unlikely to explain the magnitude of change seen. Recent studies of the lumbar fascia have shown that it can transmit loads and has contractile properties and hence is an intriguing candidate for the origin of these forces[50,51]. Further studies of this structure in scoliosis would therefore be of interest.

### Pressure profiles

As well as differences in pressure levels, there were also profound qualitative differences between the pressure profiles measured in the study scoliotic discs during surgery (Figure 4 and 5) and those found in previous measurements made in non-scoliotic discs of comparable age[25] or in healthy animal discs [28,52]. In non-degenerate discs, a hydrostatic region of constant pressure, 'the functional nucleus' is found across most of the disc apart from the first few millimetres of the outer annulus where stresses fall steeply[25]. No such profile was seen in the scoliotic discs despite their young age. Many of the profiles measured had characteristics previously seen only in degenerate [26]or asymmetrically loaded discs[53,54]such as annular stress concentrations or non-hydrostatic nuclei. The non-scoliotic but pathological discs in this study also showed stress peaks and evidence of stress gradients in the sagittal plane (Figure 10).

### Stress peaks and asymmetrical loading

The origin of disc stress peaks has been discussed previously in the literature[25,27] in non-scoliotic discs. In cadaveric discs, annular stress peaks seen in degenerate discs are thought to be due to depressurisation of the nucleus and increasing compressive loading of the annulus. Flexion or extension of discs often caused stress peaks to develop. Interestingly, some mildly degenerate discs developed annular stress peaks after depressurisation resulting from fluid loss after creep loading (1200 N over 3 hrs); this fluid loss also exacerbated the effects of flexion/extension. In the study scoliotic discs, although depressurisation was not seen in the hydrostatic region, the abnormal profiles suggest that the annulus was relatively dehydrated thus possibly leading to the stress peaks seen. If these pressures are present in daily life, these discs may be subjected to increased levels of creep loading on a daily basis in addition to the scoliotic lateral flexion and rotation deformity. Other extrinsic influences such as imposed torsion during positioning could also lead to the stress peaks observed, since combined flexion and torsion is reported to produce high stresses in the outer regions of postero-lateral annulus[53,54] possibly induced by resistance of the annulus fibres to torque[55]. If the stress profile is indeed affected by resistance of the annulus fibres to torsion or to other imposed deformations because of the abnormal lamellar organisation in scoliotic discs[40,56], results on tests from non-scoliotic discs however may not predict how stresses will be altered in scoliosis.

In 18/24 discs, higher stresses were found on the concave side of the curve compared to the convex indicating asymmetrical loading. This is obviously not explainable by asymmetrical muscle loading since the spinal muscles were relaxed in these patients. The simple presence of concave annular stress peaks, due to the possible previously mentioned factors, could lead to a stress gradient across the disc. However, even in some discs without a defined stress peak, asymmetrical loading was present.

## Conclusion

This study has found that stresses in scoliotic discs are abnormal. Scoliotic discs in recumbent anaesthetised, muscle relaxed patients have higher nuclear hydrostatic pressures than those measured in non-scoliotic discs in this study and reported in the literature. In 18/24 discs from patients, a stress gradient from concave to convex sides of the disc was measured, with some very high differential stresses (*c*. 1.0 MPa, Figure 9), indicating asymmetrical loading. In addition, the stress profiles seen in the scoliotic discs were very different to normal discs, showing similarities found in previous studies of degenerate discs [25-27].

Without the presence of muscle activity, these findings are very surprising and intriguing. Previous studies have implied that asymmetrical muscle loading is the origin of asymmetrical loading which via changes in cellular activity and hence asymmetrical growth causes a "vicous cycle" of progression in an otherwise physiologically normal spine[57,58]. The results shown indicate the presence of abnormal and asymmetric stresses in the scoliotic disc but in this case not due directly to muscle action. If they arise from remodelling of disc and ligaments or are due to alterations in thoracolumbar fascial mechanics they are likely to be carried through to normal daily postures. This could influence cellular behaviour and growth and hence generate progression of the scoliotic deformity.

## Authors' contributions

AM carried out the pressure measurements in vivo, analysed the data and drafted the manuscript. DJ and MS were responsible for setting up and validating the pressure measurements in vitro and providing technical help. JF provided the clinical overview and expertise. JU and JF conceived the study, were involved in the study design and co-ordination and helped draft the manuscript. All authors read and approved the final manuscript.
